# Structural insights into the activation initiation of full-length mGlu1

**DOI:** 10.1007/s13238-020-00808-5

**Published:** 2020-12-05

**Authors:** Jinyi Zhang, Lu Qu, Lijie Wu, Xiaomeng Tang, Feng Luo, Weixiu Xu, Yueming Xu, Zhi-Jie Liu, Tian Hua

**Affiliations:** 1 iHuman Institute, ShanghaiTech University, Shanghai, 201210 China; 2 School of Life Science and Technology, ShanghaiTech University, Shanghai, 201210 China; 3 University of Chinese Academy of Sciences, Beijing, 100049 China; 4 CAS Center for Excellence in Molecular Cell Science, Shanghai Institute of Biochemistry and Cell Biology, Chinese Academy of Sciences, Shanghai, 200031 China

**Dear Editor,**

Glutamate is the main excitatory neurotransmitter in the human brain, and it exerts diverse responses through ionotropic glutamate receptors (iGluRs) and metabotropic glutamate receptors (mGluRs) (Nakanishi and Masu, 1994). mGluRs are members of the C family of GPCRs, and are divided into three groups based on G protein coupling, sequence homology, and ligand selectivity (Stansley and Conn, 2019). mGlu1 and mGlu5 belong to group I and predominantly couple to Gq/11. They are postsynaptic glutamate receptors that respond much slower than the typical postsynaptic AMPA or NMDA-type iGluRs (millisecond timescale) (Scheefhals and MacGillavry, 2018). mGlu1 is involved in multiple physiological processes in the central nervous system (CNS) and is a promising therapeutic target for treating CNS associated disorders. Notably, positive allosteric modulators (PAMs) of mGlu1 show the selectivity in the striatum dopamine signaling inhibition and hold the potential for treating schizophrenia with fewer side effects (Stansley and Conn, 2019).

Constitutive homo- or hetero-dimerization is the defining feature of class C GPCRs, and mGluRs form homodimers mediated by the intermolecular disulfide bond within the N-terminal extracellular domain (ECD) (Wu et al., 2014). The large ECD of mGluRs can be divided into two parts: the venus flytrap (VFT) domain and the cysteine-rich domain (CRD) (Muto et al., 2007). In contrast to GPCRs of other classes, ligands bind to the orthosteric site in the VFT domain or the allosteric site in the 7TM domain (Pin and Bettler, 2016).

So far, crystal structures have been determined for the orthosteric ligand bound (agonist or antagonist) and ligand free form of VFT domain of mGlu1 (Kunishima et al., 2000; Tsuchiya et al., 2002), and the negative modulator (NAM) bound 7TM domain of mGlu1 (Wu et al., 2014). These structures provide clues about the dimerization of the VFT domain and high-resolution architecture of the mGlu1 transmembrane region. However, the full-length structure of mGlu1 is still unknown and the agonist-induced conformational transition of mGlu1 remains elusive due to the missing apo and active full-length mGlu1 structures. Here, we present two full-length mGlu1 structures in the apo and intermediate active states at 3.96 Å and 3.65 Å, respectively, using cryo-EM single particle analysis. This study captures a new intermediate state of mGluRs and provides additional insights into the dynamic activation process of mGluRs.

To enhance the expression and stability of mGlu1, the wild-type mGlu1 was modified by N- and C-terminal truncations and the optimized construct was expressed in the insect cell system (See Supplementary Materials). To seek the mGlu1 active conformation, the orthosteric agonist L-Quisqualic acid together with PAM Ro 67-4853 were added during protein purification. L-Quisqualic acid is a group I mGluRs preferred agonist that shows higher potency than glutamate (Schoepp et al., 1999), and Ro 67-4853 is a selective PAM of mGlu1 (Knoflach et al., 2001). Eventually, we obtained the cryo-EM structure of the full-length mGlu1 homodimer at a global resolution of 3.96 Å (Fig. S1, Table S1, Supplementary Materials). Similar to the full-length mGlu5, the CRD, VFT and 7TM domains constitute the whole homodimer conformation of mGlu1 with central symmetry (Fig. 1A).

**Figure 1:**
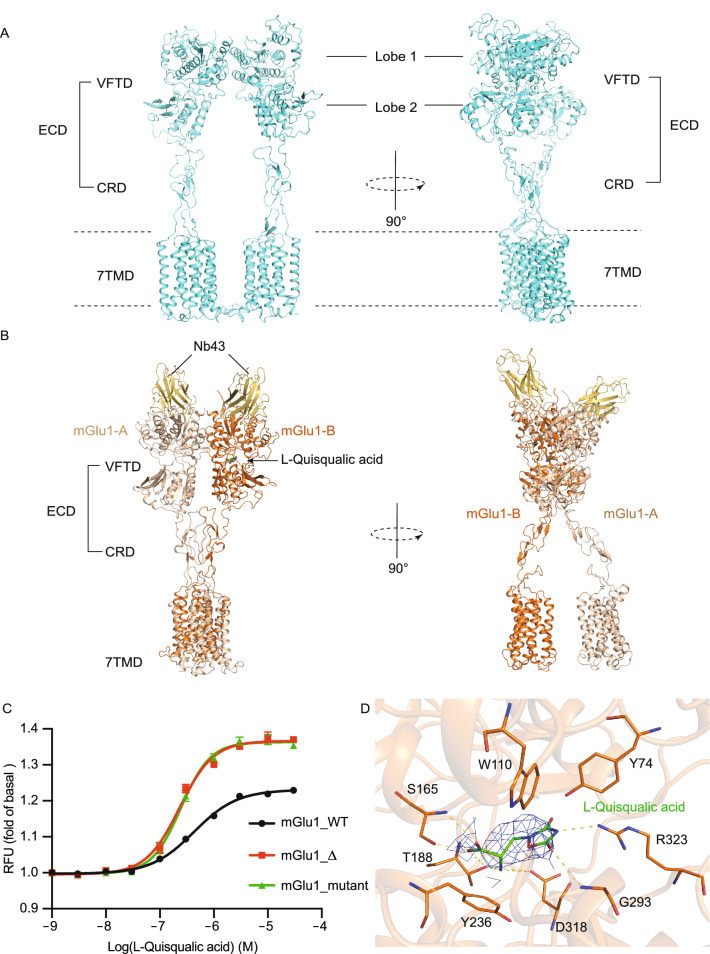
**Cryo-EM structures of full-length mGlu1 in apo and intermediate state**. (A) Overall structure (left) and side view (right) of apo state mGlu1. (B) Overall structure (left) and side view (right) of Nb43-bound mGlu1. The color codes are: Nb43 (yellow), ligand free subunit (wheat) and named as mGlu1-A, L-Quisqualic acid (green sticks, agonist)-bound subunit (orange), referred to here as mGlu1-B. (C) Effects of mGlu1 wild-type (mGlu1_WT, black) constructs for cryo-EM (apo construct mGlu1_△, red; Nb43-bound construct mGlu1_mutant, green) on the agonism of L-Quisqualic acid. Fluorescence intensity of intracellular calcium is represented by the relative fluorescence units (RFU). Data are mean ± s.e.m. (*n* = 3). (D) The binding pocket of L-Quisqualic acid in mGlu1. L-Quisqualic acid is shown as green sticks and side chains of key interaction residues are shown as orange sticks. Cryo-EM density map for L-Quisqualic acid is shown as blue

Generally, the VFT domain contains the orthosteric binding site and shows two major conformational states: open or closed state. An open state is when the VFT domain is in inactive state in presence or absence of ligand, while a close state is induced by agonists, which may lead to receptor activation (Kunishima et al., 2000; Tsuchiya et al., 2002). However, the activation of mGlu receptors from a resting state (apo or inactive state) to an active state requires additional large conformation changes of both receptors in homodimer. In the resting state, the orientations of lobe 2s in the two VFTs are distant, while in the active state, lobe 2s rotate and become closer (Kunishima et al., 2000). Based on the solved crystal structures, there exist several different conformation combinations of VFTs in mGluR dimers: “Roo” (rest open-open), “Rcc” (rest close-close), “Aoo” (active open-open), “Acc” (active close-close), and “Aco” (active close-open) (Doumazane et al., 2013).

Although an excess amount of agonist and PAM were added during mGlu1 purification, in the cryo-EM structure, there is no electron density either for L-Quisqualic acid in the ligand binding pocket of VFTs, or for PAM Ro 67-4853 in 7TM domains, indicating that mGlu1 is in the ligand free form. Compared to the crystal structure of the ligand free VFT domain of mGlu1 (PDB code 1EWT) (Kunishima et al., 2000), conformation of the VFT domain in the full-length mGlu1 is in the “Roo” state (Fig. S2A and S2B). Furthermore, our full-length mGlu1 structure is almost identical to the inactive (apo) structure of full-length mGlu5 (PDB code 6N52) (Koehl et al., 2019), so we conclude that our mGlu1 structure is in apo form (Fig. S2C).

According to previous reports on mGlu1 structures, lobe 1 from each VFT constitutes the dimmer interface and it has been proposed that an intermolecular disulfide bridge of C140 connects lobe 1s (Kunishima et al., 2000). In our full-length mGlu1 structure, residue Cys140 lies in the disordered region of VFTs and the disulfide bond is invisible, while the dimer interface is also formed by the B and C helices in VFTs (Fig. S2C). In addition, the distance between two lobe 2s is quite far. Therefore, the connected CRD and 7TM are also relatively far from each other (Figs. 1A and S2C).

To further explore the activation process of mGlu1, we learned that the nanobody Nb43 acts as a selective PAM of mGlu5 and is able to activate mGlu5 together with orthosteric agonist L-Quisqualic acid and PAM CDPPB (Koehl et al., 2019). Since the VFT domain of mGlu1 shares high sequence homology (70%) with mGlu5 (Fig. S3), Nb43 is tried to stabilize full-length mGlu1 after mutating seven Nb43 binding residues in the VFT of mGlu1 to the corresponding residues in mGlu5. The functional assay showed that the mutated mGlu1 can still be activated by L-Quisqualic acid, indicating that the mutations do not affect mGlu1’s binding with L-Quisqualic acid nor its signaling. Eventually, we solved the cryo-EM structure of Nb43-bound mGlu1 at global resolution of 3.65 Å (Figs. S1, 1B, 1C and Table S1).

In the Nb43-bound mGlu1 structure, although the same nanobody and othosteric agonist of mGlu5 are used, significant structural differences between these two receptors are observed. The active conformation of mGlu5 shows C2 symmetry (PDB code 6N51), while the mGlu1 dimer is asymmetric (Fig. S4A). This may be caused by agonist L-Quisqualic acid only binding to one of the VFTs and causing its closed state, which is henceforth referred to as mGlu1-B (Figs. 1D, S4E and S4F). However, the other VFT is in open state (mGlu1-A), even though Nb43 binds to both monomers of the dimeric mGlu1. The structure of mGlu1-A is similar to mGlu1 in apo state (Fig. S2D and S2E). For mGlu1-B, the agonist induces the VFT to the closed form and results in the movement and rotation of the CRD and 7TM domains, similar to that of a monomer in the active mGlu5 structure (Fig. S4A–D). Therefore, this Nb43-bound full-length dimeric mGlu1 is in the “Aco” conformation.

In the closed VFT domain, the binding position of L-Quisqualic acid is near the glutamate binding site in the VFT (PDB codes 1EWK and 1ISR) (Fig. S4E). L-Quisqualic acid interacts with both lobe 1 and lobe 2, and thus induces the closure of the VFT domain. Different from glutamate, L-Quisqualic acid contains a heterocyclic ring that forms a π - π interaction with Trp110. In addition, the ester group forms a hydrogen bond with Arg323 (Fig. 1D). The binding mode of L-Quisqualic acid in mGlu1 is similar to that in mGlu5 (Fig. S4F). Interestingly, our L-Quisqualic acid-bound VFT domain structure is almost identical to the crystal structure of glutamate-bound VFT domain of mGlu1 (PDB code 1EWK), and both are in the “Aco” conformation (Fig. S5). Each VFT binds with one Nb43, and their interactions are mainly between the complementarity determining regions 2 and 3 (CDR2 and CDR3) of the nanobody and the two loops (N362-E370 and R379-S388) of VFTs (Fig. S6). In addition, the B-C intersubunit interface in VFT of the Nb43-bound mGlu1 shows conformational change from apo state (Fig. S6A–C).

The observed “Aco” conformation, rather than the expected “Acc” conformation, in this Nb43-bound full-length mGlu1 structure indicates that othosteric agonist binding plays an essential role in the activation and conformational transition of mGlu1. Nb43 serves as a PAM, which assists L-Quisqualic acid binding but is not potent enough to fully activate the receptor. This result is consistent with the functional activity of Nb43 for mGlu5 (Koehl et al., 2019). Previous studies have also illustrated that the othosteric agonist is able to bind to both VFTs, and that the closure of VFT is the first step of the activation process for mGluRs (Kunishima et al., 2000; Bessis et al., 2002; Tsuchiya et al., 2002; Tateyama et al., 2004; Huang et al., 2011; Koehl et al., 2019).

In the apo state of mGlu1, the distance between the Cα atoms of Val523-Val523 and Asn235-Asn235 from the two lobe 2s of VFTs are 60 Å and 30 Å, respectively. Conversely, the corresponding distances in the L-Quisqualic acid-bound mGlu1 structure is reduced to 33 Å and 12 Å (Fig. 2A and 2B). In addition, the distances between the two CRDs and 7TM domains are also reduced from the apo state to the Nb43-bound state. These compactions of the mGlu1 structure are due to the dramatic movement of agonist-bound mGlu1-A around mGlu1-B (Fig. 2A and 2B). However, the agonist-induced conformational change of mGlu1 is less than that of mGlu5 (Koehl et al., 2019). For example, the buried surface areas between Nb43-bound mGlu1 and active mGlu5 are 1,312 (Fig. S6D) and 2,116 Å^2^, respectively. Based on these results, we conclude that our mGlu1 structure is in an intermediate state during mGlu1 activation. Moreover, based on the crystal structures of the VFT domain of mGlu1, there exists a series of conformational changes going from “Roo” to “Aco” to “Acc” upon agonist binding (Kunishima et al., 2000; Tsuchiya et al., 2002). This observation in the full-length mGlu1 structures also indicates the dynamic and complex activation of mGluRs. The “Acc” state of mGlu1 should be obtained after agonists binding at both VFTs and large conformational changes of VFT to 7TM domains are also expected (Fig. 2E).

**Figure 2:**
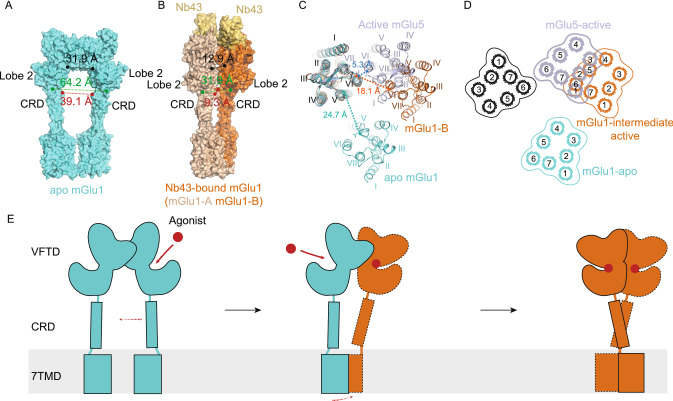
**Conformational transition of VFT, CRD and 7TM of mGlu1 in different states**. (A and B) Intersubunit distance of Nb43-bound structure (B) is closer than that of the apo state structure (A). (C) Comparison of 7TM domains in apo state mGlu1, Nb43-bound mGlu1, and active mGlu5. The mGlu1-B and one monomer from active mGlu5 are aligned to one monomer of apo state mGlu1 for reference. (D) Schematic of the rearrangement of 7TM domains with color codes as in (C). (E) A proposed model of conformational change during mGlu1 activation. Agonist, red dot; agonist-bound subunit, orange; Apo subunit, cyan. Conformation change after agonist binding is indicated by dashed red arrow

The orthosteric binding pockets of mGluRs are located in their VFT domains. The agonist binding results in the transduction of activation signals through VFT, CRD and 7TM domains to the downstream effector proteins. However, in our structure, only mGlu1-B is in the agonist-bound form, while mGlu1-A is in the apo form. However, the manner in which this diversity in the VFT domains impacts the 7TM domain is intriguing.

When analyzing the conformational changes of 7TMs from the apo- to agonist-bound states of mGlu1, the 7TM domain of mGlu1-B rotates into the proximity of 7TM of mGlu1-A by spinning around 70 degrees and moving 6 Å (Fig. 2C) relative to mGlu1-A. Figure 2C and 2D show the relative orientation and location of the 7TM domains of mGlu1 and mGlu5 structures when other corresponding 7TM domains are superimposed onto the 7TM domain of mGlu1-A. In the above transition, the closest distance between two 7TM domains in apo form is 24.7 Å, which is between two TM5-Cα of N750^5.37^. After the transition, it decreases to 18.1 Å between mGlu1-A-TM6-Cα of I804^6.56^ and mGlu1-B-TM6-Cα of I804^6.56^ in the agonist-bound form. In the mGlu5 structure, the smallest distance of 5.3 Å is between the corresponding Cα of TM6 in the active form (Fig. 2C). This result shows that our agonist-bound mGlu1 structure is indeed in the intermediate state, in the process of transforming to the active state (Fig. 2E).

Moreover, PAM Ro 67-4853 is reported to interact with the 7TM domain (Knoflach et al., 2001) and modulate mGlu1 into active state together with agonist L-Quisqualic acid. However, similar to mGlu5, there is no obvious electron density for the PAM at the allosteric site in the 7TM domain. In comparing the structural difference between the 7TM domains in Nb43-bound mGlu1 and active mGlu5 structures, the root mean square deviation (rmsd) of Cα atoms between active mGlu5 and mGlu1-A and mGlu1-B is 2.1 Å and 1.7 Å, respectively, indicating that mGlu1-B is more similar to the active state than that of mGlu1-A.

The apo and intermediate active states of full-length mGlu1 structures captured in this study provide structural snapshot of mGluRs activation process. This intermediate state structure of mGlu1 unveils that the agonist induced orientation change of the VFT domain is the key step in initiating the activation of mGlu1 receptor, more interestingly, each monomer can be activated independently. Previous studies show that postsynaptic glutamate receptors respond to ligands much slower than that of the typical postsynaptic AMPA or NMDA-type iGluRs in the neurotransmitter signaling. Our study provides the structure evidence that the activation of mGluRs involves time consuming sequential mechanical movements and coordination of VFT, CRD and 7TM domains, while transmitting signals through channels is a much more straightforward and faster process. Thus, the structure difference provides the diversity of glutamate receptors in responding to stimulus.

## Electronic supplementary material

Below is the link to the electronic supplementary material.

Supplementary material 1 (PDF 2282 kb)
