# Supplementary material for: Structural insights into the activation initiation of full-length mGlu1
Source: Protein Cell. 2020 Dec 5;12(8):662–7. doi: 10.1007/s13238-020-00808-5 (PMC8310541; doi:10.1007/s13238-020-00808-5)
Supplement: Supplementary file 1 — Supplementary material 1 (PDF 2282 kb) [file 13238_2020_808_MOESM1_ESM.pdf]

Supplemental materials

Materials and methods

Constructs design and expression

To improve the expression of the receptor in the cryo-EM construct of the full length mGlu1, the human wild-type mGlu1 was modified by the truncation of N-terminal residues 1-30 and C-terminal residues 864-1194. The optimized mGlu1 gene was cloned into pFastBac 1 vector with the N-terminal 10× His tag, the haemagglutinin (HA) signal peptide followed by a FLAG-tag, and a C-terminal PreScission protease cleavage site followed by 10× His tag. mGlu1 was expressed in *Spodoptera frugiperda* Sf9 insect cells using the Bac-to-Bac Baculovirus Expression System (Invitrogen). The cells were cultured at 27 °C, harvested 48h after infection, and stored at −80 °C for future use.

The mGlu1 mutant was generated using the above modified construct in order to bind with the nanobody Nb43. Seven mutations were introduced, including T363H, P369Q, P381E, H383F, L384P, L385Q, and P388S. This mGlu1 mutant construct was also expressed in Sf9 insect cells, which were cultured at 27 °C and harvested 48h after infection.

Purification of mGlu1

Frozen cell pellets were lysed in hypotonic buffer of 10 mM HEPES (pH 7.5), 10 mM $MgCl_2$, and 20 mM KCl with EDTA-free complete protease inhibitor cocktail tablets (Roche). Raw membranes were isolated by ultracentrifugation for 35 min. Then, they were purified by washing and ultracentrifugation three times using high osmotic buffer of 10 mM HEPES (pH 7.5), 1.0 M NaCl, 10 mM $MgCl_2$, and 20 mM KCl, with EDTA-free complete protease inhibitor cocktail tablets. The purified membranes were incubated in the presence of 50 μM L-Quisqualic acid (Tocris) and 50 μM Ro 67-4853 (Tocris) at 4 °C for 1 h. For the mGlu1 mutant sample, 10

ug ml^{-1} Nb43 was additionally added. The membranes were incubated with 2 mg ml^{-1} iodoacetamide (Sigma) for 30 min and then were solubilized with 20 mM HEPES (pH 7.5), 1% (w/v) ndodecyl-beta-D-maltopyranoside (DDM, Anatrace), 0.2% (w/v) cholesterol hemisuccinate (CHS, Sigma-Aldrich), and 750 mM NaCl at 4 °C for 2.5 h. The supernatant was isolated by ultracentrifugation for 35 min, followed by incubation in TALON IMAC resin (Clontech) and 20 mM imidazole at 4 °C overnight. The resin was washed with 5 column volumes (CVs) of washing buffer I containing 20 mM HEPES (pH 7.5), 500 mM NaCl, 0.1% (w/v) DDM, 0.02% (w/v) CHS, 20 mM imidazole, 25 μM L-Quisqualic acid, and 25 μM Ro 67-4853. Washing buffer I was then gradient exchanged to washing buffer II, which contains 20 mM HEPES (pH 7.5), 100 mM NaCl, 0.01% (w/v) lauryl maltose neopentyl glycol (LMNG, Anatrace), 0.002% (w/v) CHS, 20 mM imidazole, 25 μM L-Quisqualic acid, and 25 μM Ro 67-4853. Then, the resin was washed by 10 CVs of washing buffer II. The protein was eluted by 3 CVs of elution buffer containing 20 mM HEPES (pH 7.5), 100 mM NaCl, 0.01% (w/v) LMNG, 0.002% (w/v) CHS, 250 mM imidazole, 100 μM L-Quisqualic acid, and 50 μM Ro 67-4853. The purified protein was concentrated and loaded into a Superdex 200 Increase 10/300 column (GE Healthcare) that was pre-equilibrated with 20 mM HEPES (pH 7.5), 100 mM NaCl, 0.00075% (w/v) LMNG, and 0.00015% (w/v) CHS. Peak fractions, which contain mGlu1 dimers, were pooled and concentrated to 2- 3 mg ml^{-1} for cryo-EM sample preparation.

Constructs, expression and purification of Nb43

Nb43 was synthesized and sub-cloned into a modified pET-28a (+) vector with a 6× His tag before the N-terminus. The construct was transformed into *Escherichia coli* BL21 for protein expression. Cells were grown in Luria Broth in the presence of kanamycin at 37 °C until OD_{600} reached 0.6 - 0.8. Protein expression was induced by adding 0.5 mM IPTG, and the cells were

harvested after incubation at 25 °C for 16 - 20 h. Cells were harvested by centrifugation and then resuspended in buffer A, containing 300 mM NaCl, and 40 mM Tris (pH 8.0). The supernatant was isolated by ultracentrifugation and filtration after sonication. For Nb43 purification, protocols were as follows. In brief, Ni-NTA resin was loaded to supernatant to incubate at 4 °C for 1 - 2 h with constant stirring. The resin was washed with 10 CVs of buffer B containing 200 mM NaCl, 50 mM HEPES (pH 7.5), and 50 mM imidazole. Nb43 was then eluted by 3 CVs of buffer C, containing 200 mM NaCl, 50 mM HEPES (pH 7.5), and 300 mM imidazole. Nb43 was concentrated and frozen at −80 °C for future use.

Cryo-EM sample preparation and image acquisition

A 3 µl sample of mGlu1 was applied to an alloy (Zhenjiang Lehua Electronic Technology Co. LTD, CryoMatrix® 300 mesh Mo-Au R1.2/1.3) or copper grid (Quantifoil, 300 mesh copper R1.2/1.3) with the addition of 0.05% octylglucoside (OG). A 3 µl sample of agonist-bound mGlu1 with 0.07% octylglucoside (OG) was applied to an alloy grid that was glow-discharged for 40 s using Gatan SOLARUS (950). The grids were subsequently vitrified using the Vitrobot Mark IV (Thermo Fisher Scientific) at 4 °C and 100% humidity.

For apo mGlu1, cryo-EM datasets were collected on a Titan Krios electron microscope (Thermo Fisher Scientific) equipped with a Gatan K3 summit direct electron camera (Gatan, Inc.) in super resolution mode operating at 300 kV accelerating voltage. Movies were taken under the EFTEM nanoprobe mode with a 70 µm C2 aperture and calibrated magnification of 22,500×, corresponding to a pixel size of 1.06 Å. Each movie comprises 45 frames with a total dose of 80 electrons per $Å^2$ and 3 s exposure time with a dose rate of 30 e^-/pix/s. Serial EM software (Mastronarde, 2005) was used to collect data with a defocus range of -1.0 to -2.0 µm.

For agonist-bound mGlu1, cryo-EM datasets were collected on a Titan Krios electron

microscope equipped with a Gatan K2 summit direct electron camera and a Gatan Quantum energy filter with a slit width of 20 eV in super resolution mode. Movies were taken under the nanoprobe mode, with a 50 μm C2 aperture and calibrated magnification of 130,000×, corresponding to a pixel size of 1.04 Å. Each movie comprises 45 frames with a total dose of 60 electrons per $Å^2$ and 8.1 s exposure time with a dose rate of 8 e^-/pix/s. Serial EM software (Mastronarde, 2005) was used to collect data with a defocus range of -1.0 to -2.0 μm.

Cryo-EM data processing and 3D reconstruction

CryoSPARC v.2.15 (Punjani et al., 2017) was used to analyze the datasets of apo mGlu1. 6,604 movies were collected and patch motion correction was performed. Patch CTF estimation was used to determine contrast transfer function (CTF) parameters for each non-dose micrograph. 7,322,716 particles were yielded by template based autopicking, and a subset of seven million particles was used to do three cycles of 2D classification. Four classes of initial models were generated by selecting 1,715,013 particle projections. After several rounds of 3D classification, the best model was generated and then used as the reference model for the final 3D classification. The final dataset of 134,512 particle projections was used for the final homogenous and nonuniform refinement in cryoSPARC, and a density map with a nominal resolution of 3.96 Å was determined by gold standard Fourier shell correlation (FSC) using the 0.143 criterion. Estimation of local resolution was determined with the local resolution refinement in cryoSPARC.

RELION 3.1 (Scheres, 2012) was used to analyze the datasets of agonist-bound mGlu1. 18,832 movies were collected and motion correction was performed by MotionCor2 (Zheng et al., 2017). CTFFIND 4.1 (Rohou and Grigorieff, 2015) was used to determine CTF parameters for each non-dose micrograph. 2,909,206 particles were yielded by Gautomatch v 0.56

(http://www.mrc-lmb.cam.ac.uk/kzhang/Gautomatch), and a subset of one million particles was used for 2D classification. 217,068 particles were extracted for generating the initial model. The best model was made from 58,627 particles after several rounds of 3D classification and was then subjected to the 3D refinement with C1 symmetry and Bayesian polishing. A final 3.65 Å map was obtained based on the FSC using the 0.143 criterion. Local resolution estimation was also performed with the Bsoft package (Heymann, 2001).

Model building and refinement for mGlu1 structures

The apo form of mGlu5 (Koehl et al., 2019) (PDB code 6N52), the ligand-free form of mGlu1 VFT (Kunishima et al., 2000) (PDB code 1EWT), and NAM-bound mGlu1 7TM (Wu et al., 2014) (PDB code 4OR2) were used as the starting models for model building and refinement against the electron density map of apo mGlu1. L-Quisqualic acid, Nb43-bound mGlu5 (Koehl et al., 2019) (PDB code 6N51), and glutamate-bound mGlu1 VFT (Kunishima et al., 2000) (PDB code 1EWK) were used as the initial models for agonist-bound mGlu1. Models were docked into the EM density map using Chimera (Pettersen et al., 2004). Iterative manual adjustments and rebuilding were performed in COOT (Emsley et al., 2010) and phenix.real_space_refine in Phenix (Adams et al., 2010). The model statistics were validated using MolProbity (Chen et al., 2010). Structural figures were prepared in Chimera and PyMOL (http://www.pymol.org). The final refinement statistics are provided in Supplementary Table 1. The extent to which any model was overfitted during refinement was measured by refining the final model against one of the half-maps and by comparing the resulting map versus model FSC curves with the two half-maps and full model.

Intracellular calcium mobilization assay

CHO-K1 cells were seeded at a density of 10^6 cells in 10 cm dishes overnight at 37 □ in F-

12 supplemented with 10% FBS. On the day of transfection, 3 µg DNA encoding the mGlu1 or its mutants was transfected to the cells by TransIT2020 (Mirus Bio). After 24 h, cells were trypsinized and seeded in black-sided, clear-bottomed 384-well plates (Greiner Bio-one) at a density of 15,000 cells per well.

On the day of measurement, growth medium was removed, and cells were loaded with 20 µL/well of 1x Fluo-4 Direct Calcium dye (Invitrogen) (prepared in HBSS buffer) and incubated at 37 °C in the dark. The FLIPR Tetra High Throughput Cellular Screening System (Molecular Devices) was programmed to initially take 10 readings (1 read per second) as a baseline before the addition of 10 µl of 3x drug solutions (prepared in HBSS buffer with 0.1% BSA). The fluorescence intensity was recorded for 2 min after drug addition to detect agonist activity. To measure PAM activity, cells were incubated at room temperature for 10 min followed by a second addition of 10 µL L-Quisqualic acid at a final concentration of 145 nM. Data were analyzed by nonlinear regression using GraphPad Prism 8.0.

Reference:

Adams, P.D., Afonine, P.V., Bunkoczi, G., Chen, V.B., Davis, I.W., Echols, N., Headd, J.J., Hung, L.W., Kapral, G.J., Grosse-Kunstleve, R.W., *et al.* (2010). PHENIX: a comprehensive Python-based system for macromolecular structure solution. Acta Crystallogr D Biol Crystallogr 66, 213-221.

Chen, V.B., Arendall, W.B., 3rd, Headd, J.J., Keedy, D.A., Immormino, R.M., Kapral, G.J., Murray, L.W., Richardson, J.S., and Richardson, D.C. (2010). MolProbity: all-atom structure validation for macromolecular crystallography. Acta Crystallogr D Biol Crystallogr 66, 12-21.

Emsley, P., Lohkamp, B., Scott, W.G., and Cowtan, K. (2010). Features and development of Coot. Acta Crystallogr D Biol Crystallogr 66, 486-501.

Heymann, J.B. (2001). Bsoft: image and molecular processing in electron microscopy. J Struct Biol 133, 156-169.

Koehl, A., Hu, H., Feng, D., Sun, B., Zhang, Y., Robertson, M.J., Chu, M., Kobilka, T.S., Laeremans, T., Steyaert, J., *et al.* (2019). Structural insights into the activation of metabotropic glutamate receptors. Nature 566, 79-84.

Kunishima, N., Shimada, Y., Tsuji, Y., Sato, T., Yamamoto, M., Kumasaka, T., Nakanishi, S., Jingami, H., and Morikawa, K. (2000). Structural basis of glutamate recognition by a dimeric metabotropic glutamate receptor. Nature 407, 971-977.

Mastronarde, D.N. (2005). Automated electron microscope tomography using robust prediction of specimen movements. J Struct Biol 152, 36-51.

Pettersen, E.F., Goddard, T.D., Huang, C.C., Couch, G.S., Greenblatt, D.M., Meng, E.C., and Ferrin, T.E. (2004). UCSF Chimera--a visualization system for exploratory research and analysis. J Comput Chem 25, 1605-1612.

Punjani, A., Rubinstein, J.L., Fleet, D.J., and Brubaker, M.A. (2017). cryoSPARC: algorithms for rapid unsupervised cryo-EM structure determination. Nat Methods 14, 290-296.

Rohou, A., and Grigorieff, N. (2015). CTFFIND4: Fast and accurate defocus estimation from electron micrographs. J Struct Biol 192, 216-221.

Scheres, S.H. (2012). RELION: implementation of a Bayesian approach to cryo-EM structure determination. J Struct Biol 180, 519-530.

Wu, H., Wang, C., Gregory, K.J., Han, G.W., Cho, H.P., Xia, Y., Niswender, C.M., Katritch, V., Meiler, J., Cherezov, V., *et al.* (2014). Structure of a class C GPCR metabotropic glutamate receptor 1 bound to an allosteric modulator. Science 344, 58-64.

Zheng, S.Q., Palovcak, E., Armache, J.P., Verba, K.A., Cheng, Y., and Agard, D.A. (2017).

MotionCor2: anisotropic correction of beam-induced motion for improved cryo-electron microscopy. Nat Methods 14, 331-332.

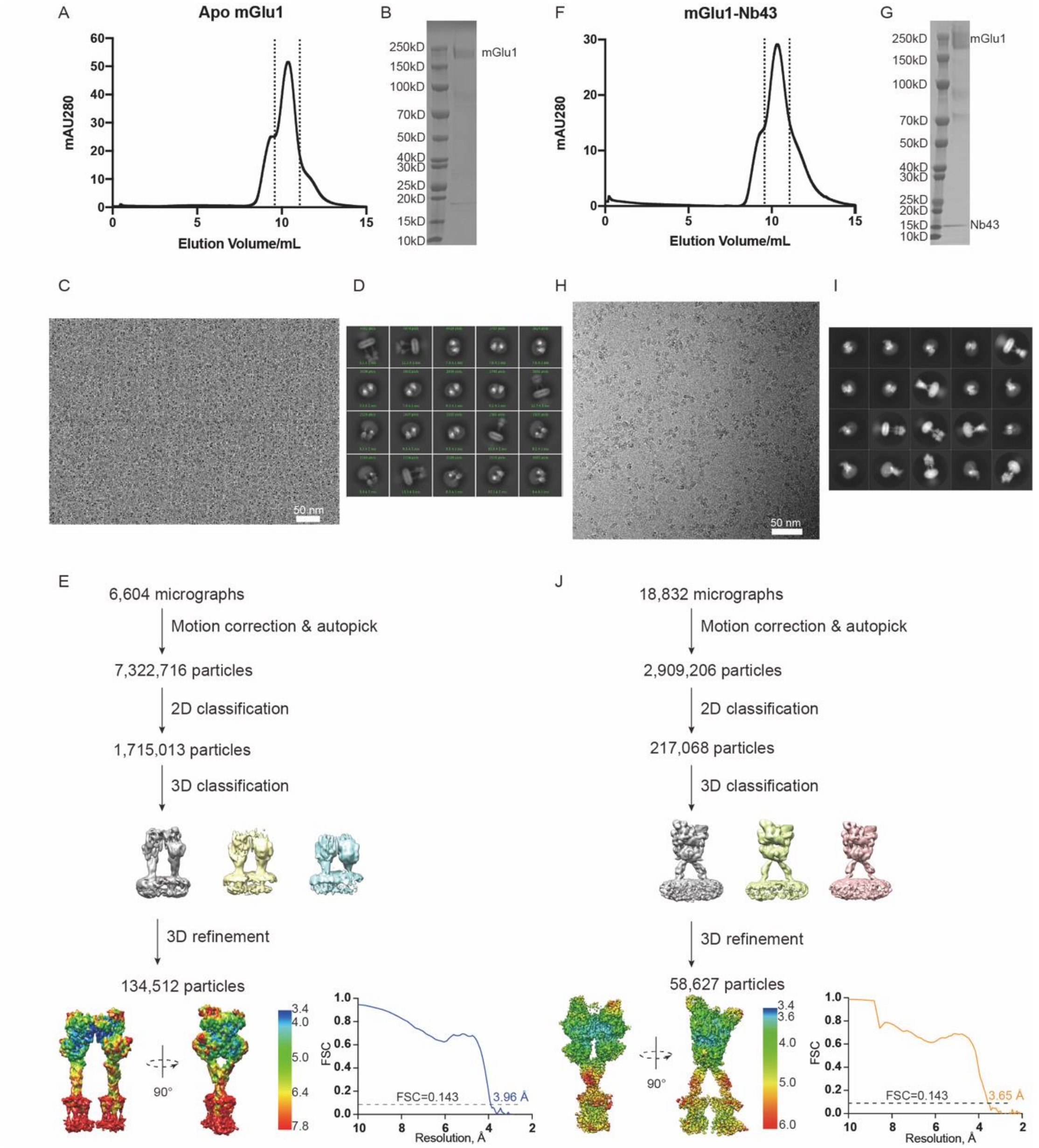

Supplementary Figure 1. mGlu1 cryo-EM sample preparation and data processing workflow. (A and F) Superdex 200 size-exclusion chromatography profiles of apo mGlu1 and Nb43-bound mGlu1. (B and G) SDS-PAGE analysis. (C and H) Representative cryo-EM images. (D and I) Representative 2D classifications. (E and J) Cryo-EM data processing workflow. Cryo-EM maps are colored by local resolution (Å). Resolutions of apo mGlu1 and Nb43-mGlu1 are 3.96 and 3.65 Å, respectively, which are indicated by Gold-standard FSC curves at FSC=0.143.

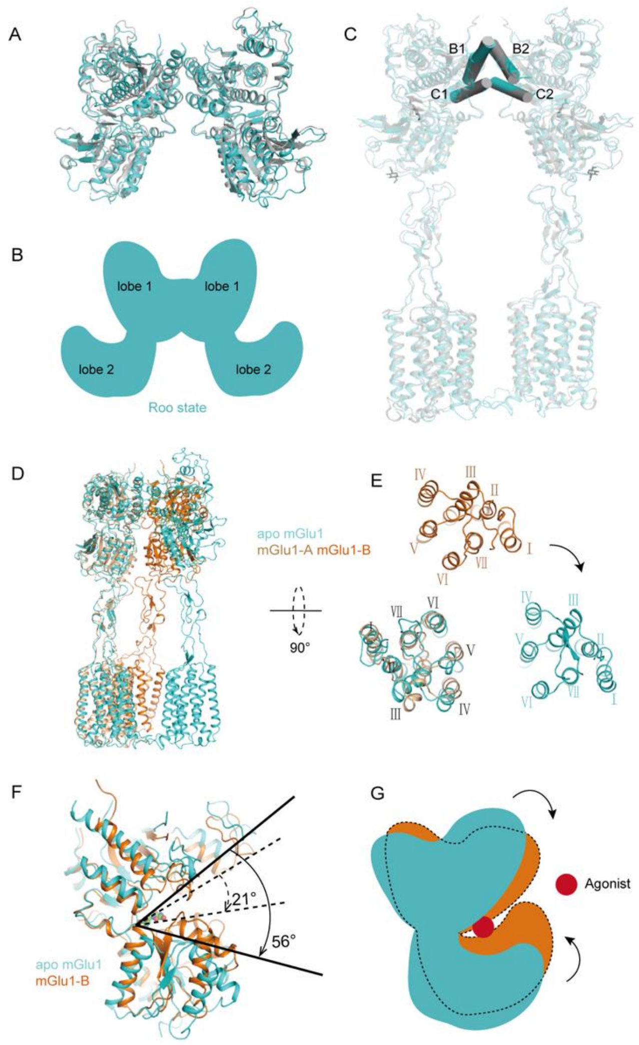

Supplementary Figure 2. Comparison of apo-state and Nb43-bound mGlu1 structures. (A) Comparison of the VFT domain in the cryo-EM structure (cyan) with the crystal structure of VFT (PDB code 1EWT) (gray), both in "Roo" conformation. (B) Schematic of the "Roo" conformation of VFT in mGlu1. (C) Overall structure comparison of apo state mGlu1 (cyan) and apo state mGlu5 (PDB: 6N51) (gray). Cylindrical helices indicate the dimer interface. (D-E) Comparison between apo (cyan) and Nb43-bound mGlu1 structures. Side view (D) and intracellular view of the 7TM domains (E). (F) Comparison between agonist-bound VFT and apo state VFT structures. Solid line gives the angle of apo VFT and the dash line give the angle of the agonist-bound VFT. (G) Schematic of the conformational transition from apo to agonist-bound

VFTs.

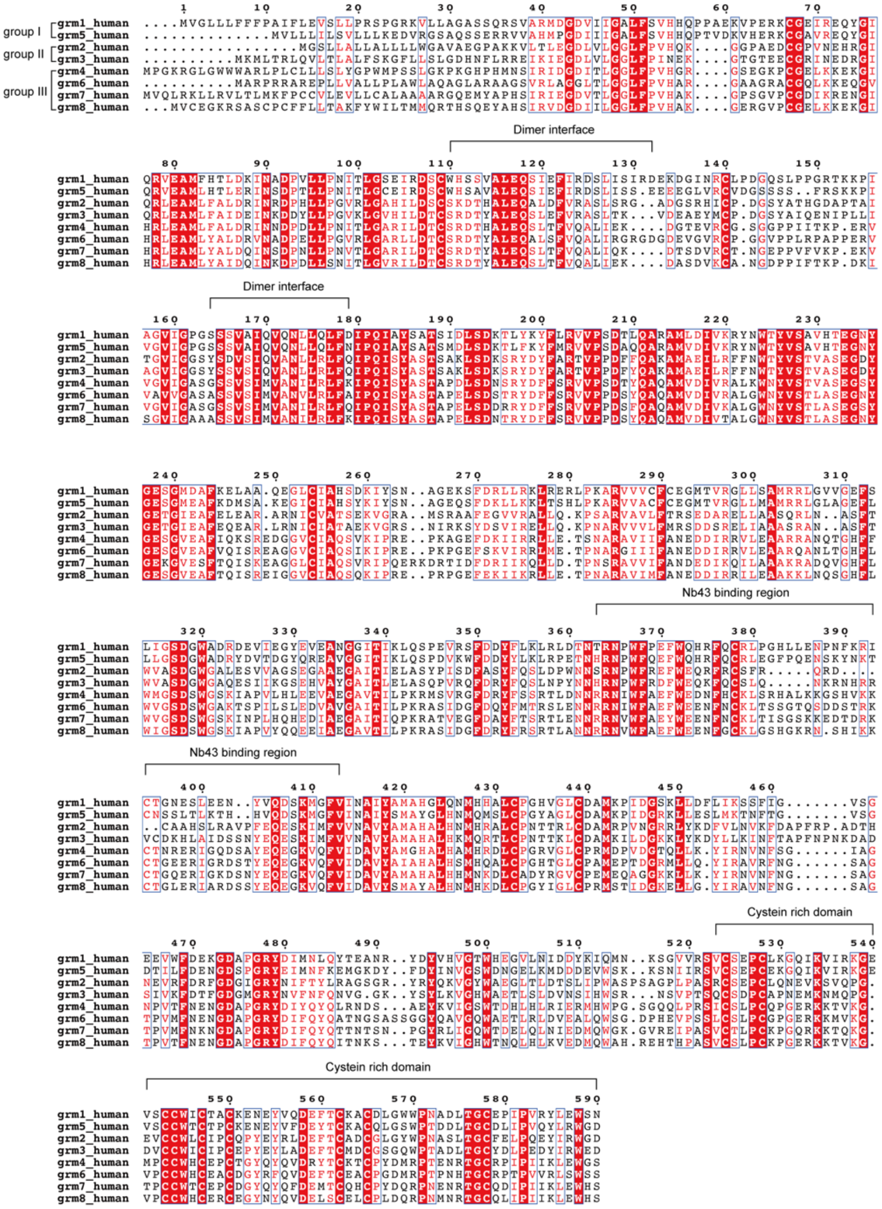

Supplementary Figure 3. Sequence alignment of the ECD of human mGluRs. Red background indicates conserved residues; red font shows residues with similar properties. The sequences involved in the dimer interface, Nb43 binding, and cysteine rich domain (CRD) are labelled. Sequence alignment was produced by CLUSTALW (https://www.genome.jp/tools-bin/clustalw) and the graph was generated by the ESPript 3.0 server (http://espript.ibcp.fr/ESPript/cgi-bin/ESPript.cgi).

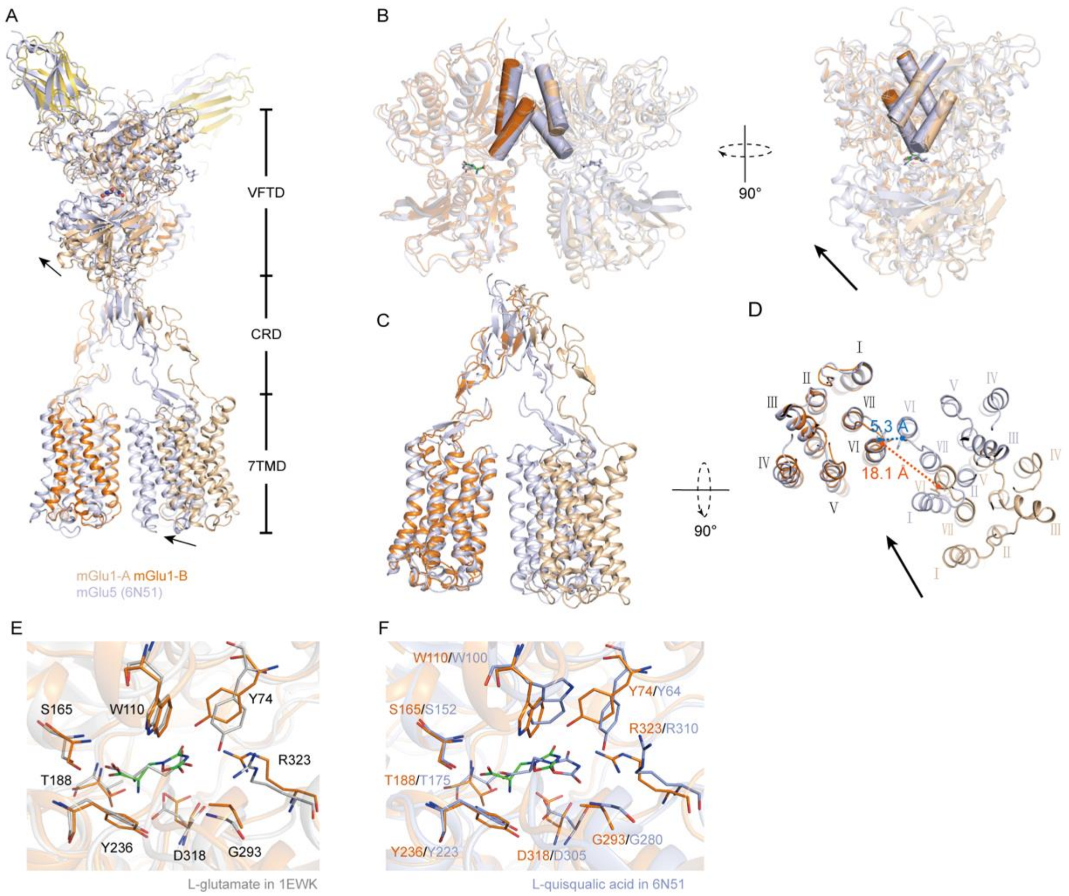

Supplementary Figure 4. Structure comparison of Nb43-bound mGlu1 and active mGlu5. (A) Overall structure comparison. mGlu1-A, wheat; mGlu1-B, orange; Nb43, yellow; L-Quisqualic acid, green. The active mGlu5 structure and L-Quisqualic acid are colored blue. (B) Comparison of VFTs. Cylindrical helices indicate the dimer interface. (C) Comparison of CRD and 7TM. (D) Comparison of the 7TM extracellular and intracellular view. Conformational transition is indicated by the black arrow. (E) Binding pose comparison of L-Quisqualic acid (green sticks) in mGlu1-B structure (orange cartoon) and L-glutamate (gray sticks) in mGlu1-VFT structure (gray cartoon, PDB: 1EWK). (F) Binding poses comparison of L-Quisqualic acid (green sticks) in mGlu1-B structure (orange cartoon) and L-Quisqualic acid (blue sticks) in active mGlu5 structure (blue cartoon, PDB: 6N51).

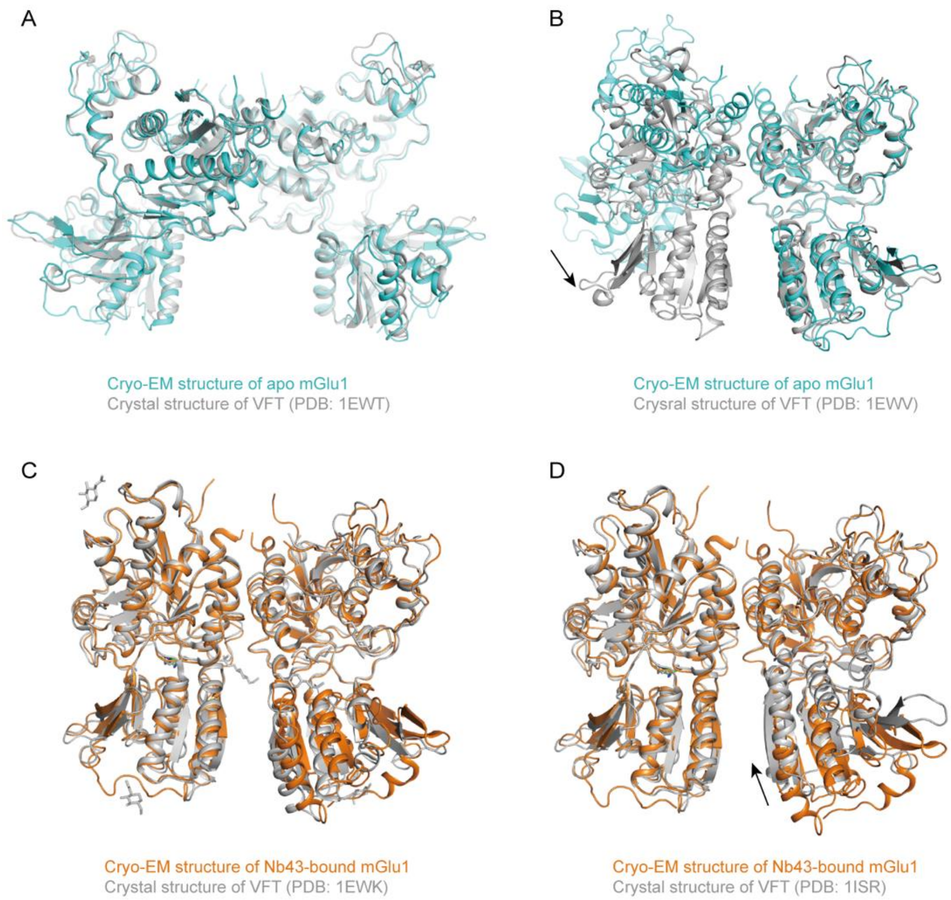

Supplementary Figure 5. Structure comparison of VFTs of mGlu1. (A) "Roo" conformation of both cryo-EM and crystal structure (PDB: 1EWT). (B) "Aco" conformation of crystal structure (PDB: 1EWV). (C) "Aco" conformation of both cryo-EM and crystal structure (PDB: 1EWK). (D) "Acc" conformation of crystal structure (PDB: 1ISR). Conformational transition is indicated by the black arrow.

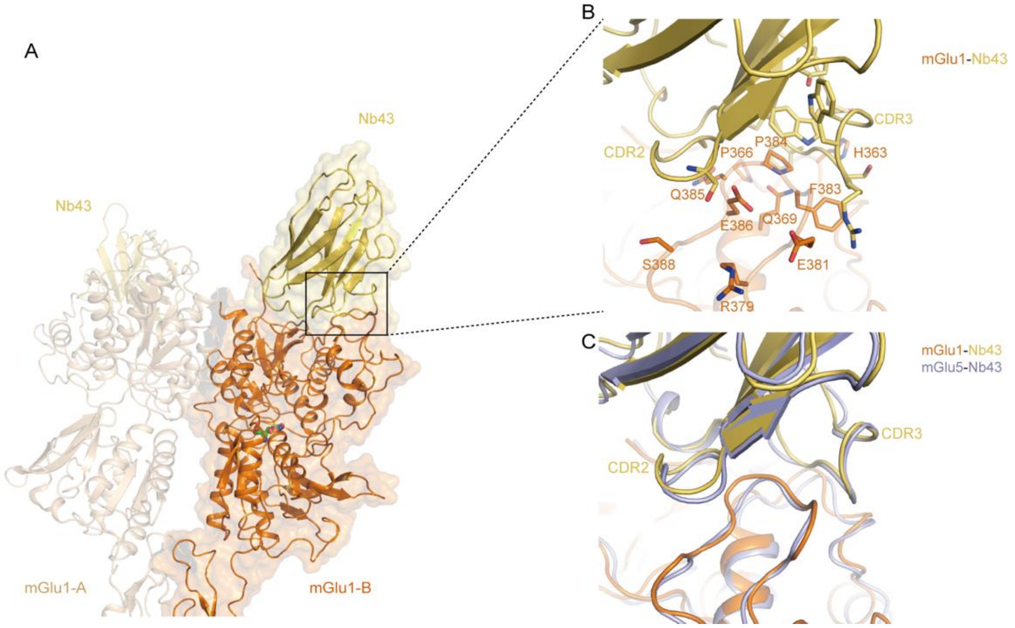

Supplementary Figure 6. Interactions between mGlu1 and Nb43. (A) Cartoon of Nb43-mGlu1. mGlu1-A, wheat; mGlu1-B with surface, orange; Nb43 with surface, yellow. (B) Interaction interface of mGlu1-B (orange) and Nb43 (yellow). Key residues from Nb43 are located at CDR2 and CDR3 and shown as yellow sticks. Key residues from mGlu1-B are shown as orange sticks. (C) Comparison of Nb43-mGlu1 (mGlu1-B, orange; Nb43, yellow) and Nb43-mGlu5 (blue) structures.

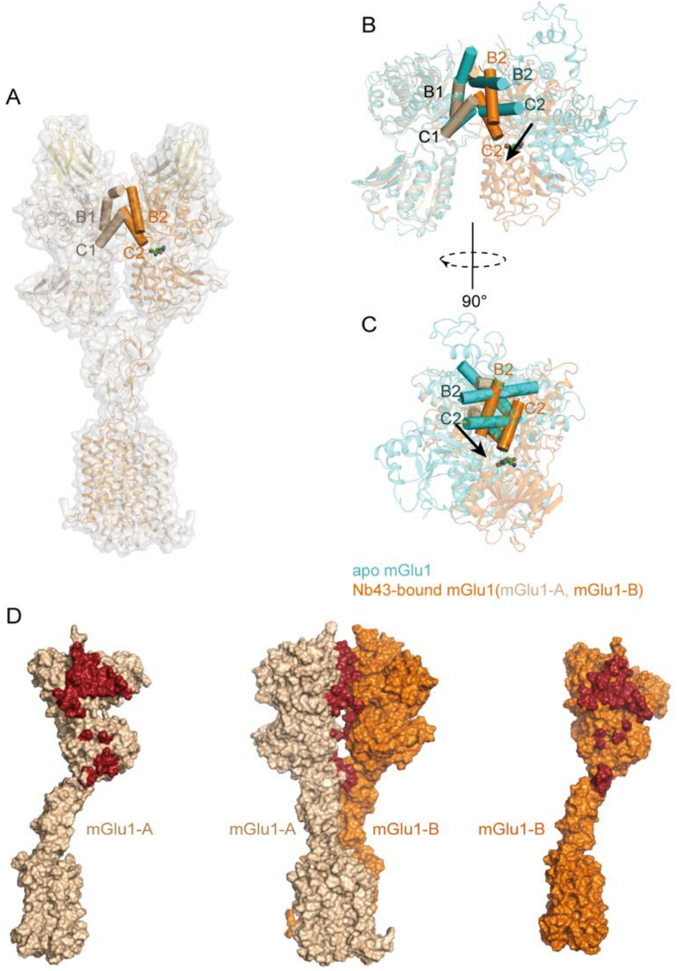

Supplementary Figure 7. Dimer interface transition after agonist binding. (A) Dimer interface within the overall structure of Nb43-bound mGlu1. Cylindrical helices (mGlu1-A, wheat; mGlu1-B, orange) show the dimer interface. (B and C) Dimer interface comparison between apo (cyan) and Nb43-bound (mGlu1-A, wheat; mGlu1-B, orange) mGlu1. The conformational change of the dimer interface is indicated by the black arrow. (D) Comparison of the intersubunit interface in the Nb43-bound mGlu1. The contact region (red) show residues within 4 Å of the opposite subunit.

Table S1. Cryo-EM data collection and refinement statistics.

	mGlu1-apo	mGlu1-Nb43
**Data collection and processing**		
Magnification	22,500	130,000
Voltage (kV)	300	300
Electron exposure (e-/Å^2)	80	60
Defocus range (μm)	-1.0~-2.0	-1.0~-2.0
Pixel size (Å)	1.06	1.04
Symmetry imposed	C2	C1
Initial particle images (no.)	7,322,716	2,909,206
Final particle images (no.)	134,512	58,627
Map resolution (Å)	3.96	3.65
FSC threshold	0.143	0.143
**Refinement**		
Initial model used (PDB code)	1EWT, 4OR2, 6N52	1EWT, 4OR2, 6N51
Map sharpening B factor (Å^2)	108.5	91.4
Model composition		
Non-hydrogen atoms	12395	14249
Protein residues	1572	1815
Ligands	0	1
B factors (Å^2)		
Protein	56.9	61.4
Ligand		
rmsd		
Bond lengths (Å)	0.01	0.01
Bond angles (°)	1.124	1.047
Validation		
MolProbity score	37.25	33.56
Clash score	2.87	2.67
Poor rotamers (%)	1.53	1.03
Ramachandran plot		
Favored (%)	84.02	85.35
Allowed (%)	15.02	14.43
Disallowed (%)	0.96	0.22
